
JOURNAL INFORMATION
==============================
NLM Title Abbreviation: ScientificWorldJournal
Journal ID: ScientificWorldJournal
NLM Journal ID: 101131163
Journal ID: TSWJ

The Scientific World Journal

ISSN: 2356-6140
EISSN: 1537-744X
Publisher: Wiley

ARTICLE INFORMATION
==============================
PMCID: PMC5901145
PMID: 17704851
DOI: 10.1100/tsw.2007.199
Article ID: 436350
Article version: 1
Subjects: Clinical Images

Gastric Signet-Ring Cell Carcinoma: Unilateral Lower Extremity Lymphoedema as the Presenting Feature

Lanznaster Gudrun 1
Adami Michele 1
Crivellaro Claudio 1
Kluge Reinhard 2
Egarter-Vigl Eduard 2
Wiedermann Christian J. 1 *
1 Division of Internal Medicine 2, Department of Medicine, Central Hospital of Bolzano, L. Böhler Street 5, I-39100 Bolzano, Italy
2 Institute of Pathological Anatomy and Histology, Central Hospital of Bolzano, L. Böhler Street 5, I-39100 Bolzano, Italy
*Corresponding author.Academic Editor: Daniel Batlle

Publication date: 2007
Electronic publication date: 2007 Aug 17
Volume: 7
First page: 1189
Last page: 1192
Copyright: Copyright © 2007 Gudrun Lanznaster et al.
Copyright year: 2007
License: This is an open access article distributed under the Creative Commons Attribution License, which permits unrestricted use, distribution, and reproduction in any medium, provided the original work is properly cited.
License URL: https://creativecommons.org/licenses/by/3.0/

Keywords: Non-pitting edema, signet ring cell carcinoma

==============================
Clinical Image
*TheScientificWorldJOURNAL* (2007) 7, 1189–1192
ISSN 1537-744X; DOI 10.1100/tsw.2007.199

TheScientificWorldJOURNAL
www.thescientificworld.com

### Gastric Signet-Ring Cell Carcinoma: Unilateral Lower Extremity Lymphoedema as the Presenting Feature

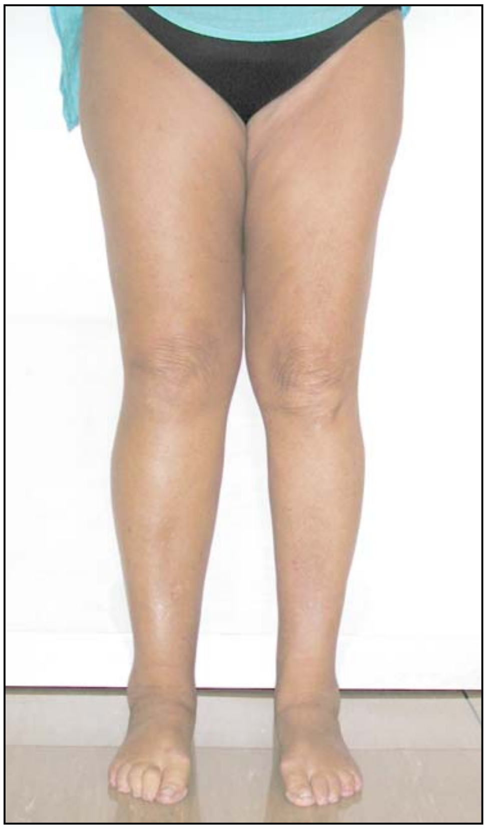

**FIGURE 1.** Mild unilateral non-pitting edema of the entire right leg.

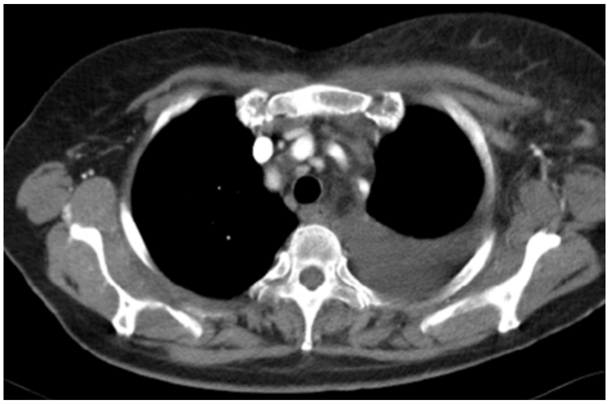

**FIGURE 2.** CT scan of the chest showing left-sided pleural effusion and paratracheal lymphadenopathy

*Corresponding author.
©2007 with author.
Published by TheScientificWorld; www.thescientificworld.com

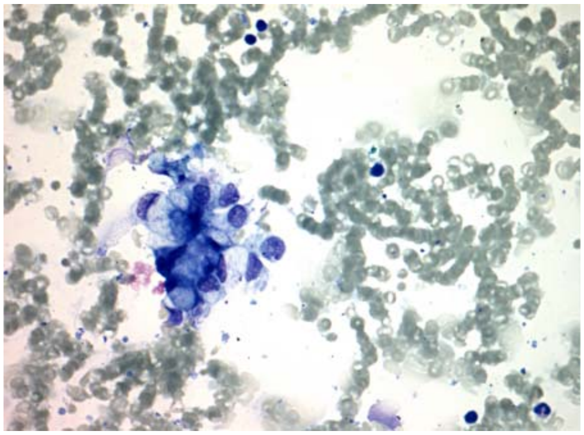

**FIGURE 3.** Fine needle biopsy of a supraclavicular lymph node showing numerous polygonal cells in a multilayered aggregate that possess intracytoplasmic vacuoles. Signet ring cells are created by a large, single vacuole with the enlarged nucleus pushed to the cell periphery. Giemsa stain 400x.

**(a)** **(b)**

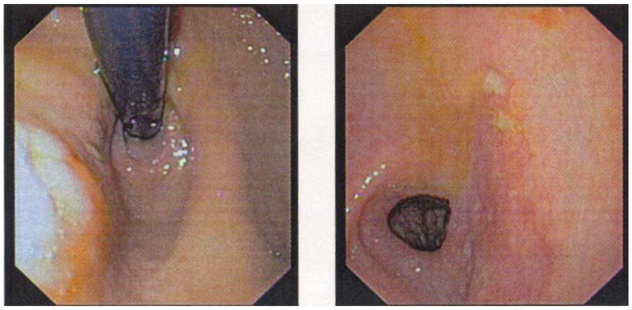

**FIGURE 4.** Upper gastrointestinal endoscopy demonstrating (a) a hiatal hernia unrelated to the leg's edema and (b) the antrum mucosal infiltration and ulceration in the stomach from which biopsies were taken (see Fig. 5).

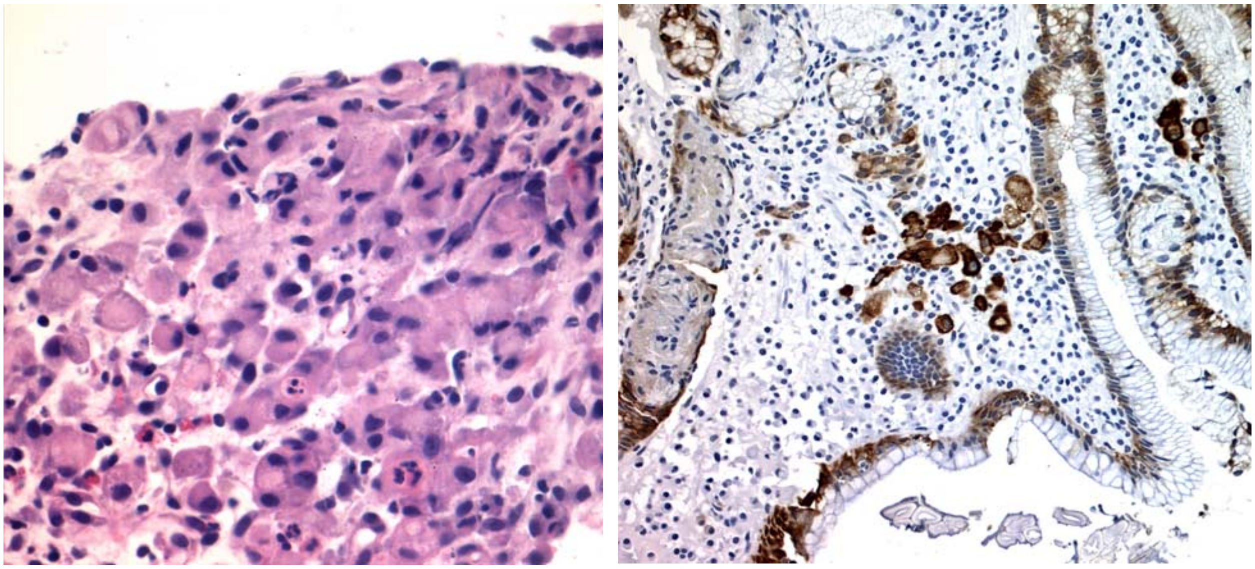

**(a)** **(b)**

**FIGURE 5.** Histology of gastric mucosal biopsies. (a) Hematoxylin-Eosin (400x) and (b) anti-cytokeratin (200x) stain of some signet ring cells of a diffuse gastric carcinoma.

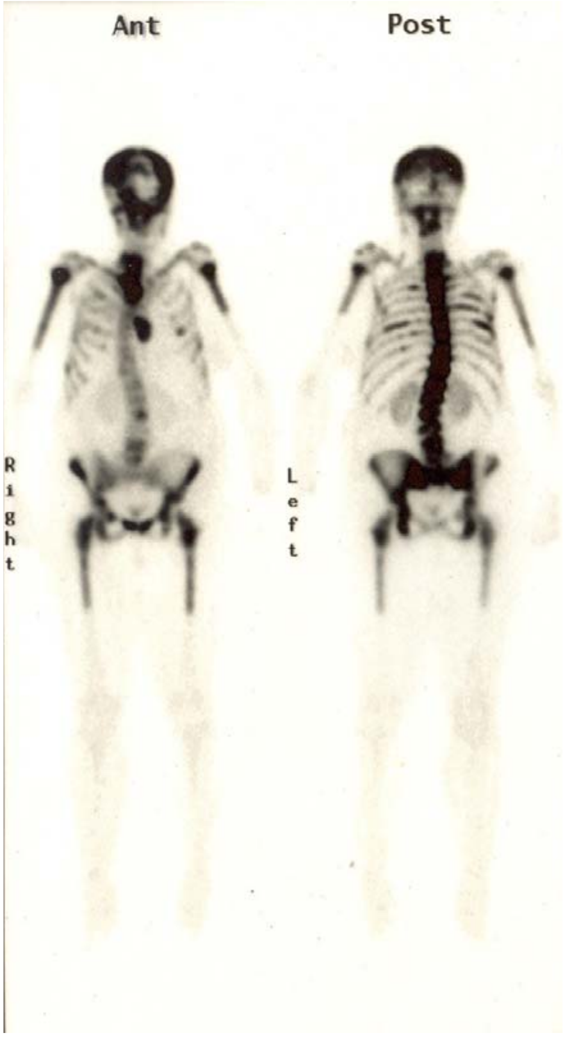

**FIGURE 6.** Bone scan with Tc-99m MDP showing multiple foci of increased isotope uptake throughout the axial skeleton compatible with bony metastatic disease

Gudrun Lanznaster[1], Michele Adami[1], Claudio Crivellaro[1], Reinhard Kluge[2], Eduard Egarter-Vigl[2], and Christian J. Wiedermann[1*]

*[1]Division of Internal Medicine 2, Department of Medicine, and [2]Institute of Pathological Anatomy and Histology, Central Hospital of Bolzano, L. Böhler Street 5, I-39100 Bolzano, Italy*

**E-mail**: christian.wiedermann@asbz.it

*Received May 17, 2007; Revised June 15, 2007; Accepted July 11, 2007; Published August 17, 2007*

**KEYWORDS:** Non-pitting edema, signet ring cell carcinoma

Persistent unilateral non-pitting edema is characteristic of venous insufficiency such as in the postphlebitic syndrome. Other acquired causes may lead to lymphoedema by lymph vessel obstruction and typically include connective tissue disease, infection, contact dermatitis and malignancy. A case of slow-onset, unilateral lymphoedema of the lower extremity is reported in whom initially no causes of peripheral lymph vessel obstruction or evidence of venous insufficiency were identified by routine laboratory testing, abdominal computerized tomography and ultrasound examination. Endoscopic examination, computerized tomography of the chest and a bone scan revealed gastric signet-ring cell cancer that had metastasized to thoracic lymph nodes and bones. Abdominal, pelvic or inguinal lymph node destruction by metastases is not a requirement for unilateral acquired lymphoedema of the lower extremity to develop in malignancy. Gastric signet-ring cell cancer may lead to non-pitting edema formation typically due to lymph system micro-metastasis. Distant site signet-ring cell malignancy should be included in the differential diagnosis of otherwise unexplained acquired peripheral lymphedema.

### REFERENCES

1. Hegemann, B., Helmbold, P., and Marsch, W.C. (2001) Genital inflammatory lymphoedema: peculiar microvascular long-distance metastasis of gastric carcinoma. *Br J Dermatol.,* **144,** 419-20.
2. Lugering, N., Menzel, J., Westermann, G., and Domschke, W. (2000) Signet ring cell carcinoma of the stomach--a case of diagnostic dilemma. *Z Gastroenterol.,* **38,** 177-80.
3. Heidenheim, M., Hansen, U., and Andersen, J. (1989) Elephantiasis-like cutaneous metastases from signet-ring cell carcinoma of the stomach. *Cutis,* **44,** 455-8.
4. Robin, P., Sicard, D., Jacob, L., Lessana-Leibowitch, M., and Cremer, GA. (1987) Scleromyxoedematous infiltrations disclosing a gastric adenocarcinoma. *Presse Med.,* **16,** 356.

**This article should be cited as follows:**

Lanznaster, G., Adami, M., Crivellaro, C., Kluge, R., Egarter-Vigl, E., and Wiedermann, C.J. (2007) Gastric signet-ring cell carcinoma: Unilateral lower extremity lymphoedema as the presenting feature. *TheScientificWorldJOURNAL* **7,** 1189-1192. DOI 10.1100/tsw.2007.199.
